# The Sodium Iodide Symporter (NIS) and Potential Regulators in Normal, Benign and Malignant Human Breast Tissue

**DOI:** 10.1371/journal.pone.0016023

**Published:** 2011-01-19

**Authors:** James Ryan, Catherine E. Curran, Emer Hennessy, John Newell, John C. Morris, Michael J. Kerin, Roisin M. Dwyer

**Affiliations:** 1 Division of Surgery, School of Medicine, National University of Ireland Galway (NUIG), Galway, Ireland; 2 School of Mathematics, Statistics and Applied Mathematics and Clinical Research Facility, National University of Ireland Galway (NUIG), Galway, Ireland; 3 Division of Endocrinology, Mayo Clinic, Rochester, Minnesota, United States of America; Cardiff University, United Kingdom

## Abstract

**Introduction:**

The presence, relevance and regulation of the Sodium Iodide Symporter (NIS) in human mammary tissue remains poorly understood. This study aimed to quantify relative expression of NIS and putative regulators in human breast tissue, with relationships observed further investigated in vitro.

**Methods:**

Human breast tissue specimens (malignant n = 75, normal n = 15, fibroadenoma n = 10) were analysed by RQ-PCR targeting NIS, receptors for retinoic acid (RARα, RARβ), oestrogen (ERα), thyroid hormones (THRα, THRβ), and also phosphoinositide-3-kinase (PI3K). Breast cancer cells were treated with Retinoic acid (ATRA), Estradiol and Thyroxine individually and in combination followed by analysis of changes in NIS expression.

**Results:**

The lowest levels of NIS were detected in normal tissue (Mean(SEM) 0.70(0.12) Log_10_ Relative Quantity (RQ)) with significantly higher levels observed in fibroadenoma (1.69(0.21) Log_10_RQ, p<0.005) and malignant breast tissue (1.18(0.07) Log_10_RQ, p<0.05). Significant positive correlations were observed between human NIS and ERα (r = 0.22, p<0.05) and RARα (r = 0.29, p<0.005), with the strongest relationship observed between NIS and RARβ (r = 0.38, p<0.0001). An inverse relationship between NIS and PI3K expression was also observed (r = −0.21, p<0.05). In vitro, ATRA, Estradiol and Thyroxine individually stimulated significant increases in NIS expression (range 6–16 fold), while ATRA and Thyroxine combined caused the greatest increase (range 16–26 fold).

**Conclusion:**

Although NIS expression is significantly higher in malignant compared to normal breast tissue, the highest level was detected in fibroadenoma. The data presented supports a role for retinoic acid and estradiol in mammary NIS regulation in vivo, and also highlights potential thyroidal regulation of mammary NIS mediated by thyroid hormones.

## Introduction

The Sodium Iodide Symporter (NIS) is a transmembrane glycoprotein found predominantly in thyroid follicular cells and mediates the active transport of iodide. This supports production of thyroid hormones Tri-iodothyronine (T_3_) and Thyroxine (T_4_), and facilitates the use of radiolabelled iodide for imaging and therapy of thyroid disease [1]. NIS is also expressed in extrathyroidal tissue but to a much lower extent. It occurs in salivary glands, small intestines, gastric mucosa, and of particular interest in this study, mammary tissue [1]. NIS is known to be naturally expressed in lactating mammary tissue to support iodide concentration into breast milk for neonatal nutrition, but is not normally detected at significant levels in nonlactating tissue [2].

Many studies have shown NIS expression in breast cancer, combined with much lower prevalence in normal, or extratumoral tissue [2], [3], [4], [5], [6], [7]. However the majority of studies on NIS expression in human tissue have been based on immunohistochemistry (IHC) which is not a quantitative technique [2], [4], [5], [6], [7]. Studies investigating NIS gene expression in malignant breast tissue provide a profile of expression which is limited due to small cohort sizes [3], [4]. Despite these limitations, it was a suggested that NIS may represent a means of identifying malignant versus non malignant tissue [2]. Functional NIS expression was shown in human malignant mammary tissue with *in vivo* iodine transport ability confirmed by scintigraphy [4], [8]. Iodide uptake has also been identified in soft tissue breast cancer metastases through scintigraphy [9]. More recently, NIS protein was shown to be detectable by IHC in brain metastases from breast cancer [10], and so the characteristic of abnormal NIS protein expression in breast tumours may be maintained in derivative metastases.

The earlier promise of the potential application of NIS as a specific indicator of malignancy has been undermined by studies which have shown NIS expression and iodide accumulation in benign (fibroadenoma) tissue [3], [6]. This is compounded by a study highlighting functional NIS expression in a fibroadenoma which was visible using a whole body gamma camera scan after administration of cytotoxic radioiodide for treatment of thyroid cancer [11].

The suitability of native functional NIS expression for treatment of breast cancer using radiolabelled iodide is also in question with the current consensus suggesting that native iodide uptake levels would be insufficient to facilitate effective treatment. Approaches have been investigated which could increase endogenous mammary NIS expression by stimulation of putative regulators, but while some data is promising [12], [13], increased knowledge of breast cancer and mammary NIS regulatory mechanisms is required.

Regulation of NIS expression in the thyroid is well understood, with Thyroid Stimulating Hormone (TSH) recognised as the major positive regulator. Negative regulation is mediated through inhibition of TSH by accumulation of T_3_ and T_4_ [1], [14]. Thyroglobulin is also a potent suppressor of NIS mRNA expression [15] and has been shown to suppress TSH-stimulated NIS activity both in vitro and in vivo [16]. Negative feedback from iodide accumulation, and cytokine mediated inhibition of NIS expression also occur [1], [14].

NIS regulation in mammary tissue has not yet been clearly established. During lactation NIS expression was shown to be optimal in the presence of oestrogen, prolactin and oxytocin [2]. In the case of breast cancer, a number of potential regulators of NIS have been identified. Retinoic acid receptor α (RARα) has been shown to increase NIS expression in MCF-7 cells in vitro [17]. Induction of functional NIS expression in breast cancer xenografts after treatment with Retinoic acid and Dexamethasone has also been demonstrated [13]. An isoform of RAR, Retinoic acid receptor β (RARβ) has been shown to promote a greater increase in NIS expression when similarly stimulated with RA in MCF-7 cells [17]. The unliganded estrogen receptor alpha (ERα) has also been shown to activate mammary NIS transcription in oestrogen receptor positive breast cancer cell lines [18]. This is supported by the presence of an oestrogen responsive element in the NIS gene promoter [18]. Phosphoinositide-3-kinase (PI3K) activation leads to increased NIS expression in MCF-7 cells and has also been shown to diminish membrane localisation of the protein in these cells [19], [20]. Although no link has been demonstrated in mammary tissue, the importance of thyroid hormone receptors in thyroidal NIS expression [1], and their differential expression in breast cancer warrants their investigation as potential mammary NIS regulators [21], [22], [23]. Previous studies of potential regulators of NIS expression in breast cancer, although informative, have been performed using breast cancer cell lines and animals models. Investigation of expression of potential regulators and their pertinence to NIS in human breast tumour tissue has not been performed.

The aim of this study was to determine the relative level of expression of NIS, and a range of receptors for putative regulators (ERα, RARα/β and THRα/β) in human breast tissue samples. Further, based on relationships observed in normal, fibroadenoma and malignant breast tissues, breast cancer cell lines were stimulated with ligands for these receptors individually and in combination, and their effect on NIS expression quantified.

## Materials and Methods

### Ethics Statement

For research involving tissue samples from human participants, ethical approval was granted by the Clinical Research Ethics Committee (University College Hospital, Galway). Written informed consent was obtained from each patient and all clinical investigation was conducted according to the principles expressed in the Declaration of Helsinki.

### Study Cohort

Breast tissue biopsies (n = 100) were obtained at the University College Hospital, Galway. The cohort consisted of 75 malignant mammary tissue biopsies, 15 normal mammary tissue biopsies harvested at reduction mammoplasty and 10 fibroadenoma biopsies. Patient demographics for this study cohort are shown in Table 1. Samples were snap frozen in liquid nitrogen immediately after excision and stored at −80°C until processed.

**Table 1:** Patient demographics of study cohort.

Patient cohort details				
	Breast Cancer	Fibroadenoma	Normal	
***No. of Subjects***	75	10	15	
**Premenopausal**	24	9	12	
**Postmenopausal**	51	1	3	
***Mean age (range)***	57 (35–90)	36 (20–50)	48 (33–78)	
**Premenopausal**	46 (35–59)	34 (20–49)	43 (33–48)	
**Postmenopausal**	62 (41–90)	50	62 (50–78)	
***Histology***	Ductal	Lobular	Other	
	58	11	6	
***Epithelial Subtype***	Luminal A	Luminal B	HER2	Basal
	31	17	13	14
***Disease Stage***	Stage 1	Stage 2	Stage 3	Stage 4
	17	35	15	8
***Disease Grade***	Grade 0	Grade 1	Grade 2	Grade 3
	4	3	23	45

### Gene expression analysis

Biobanked tissue biopsies were homogenised briefly in 1 ml TRIzol® Reagent (Invitrogen) using a Polytron PT 1600 E @ 30,000 rpm. Total RNA was extracted from malignant (n = 75), normal (n = 15) and fibroadenoma (n = 10) mammary tissue using the RNeasy Mini Kit (QIAGEN) as per manufacturer's instructions. Note: (1) TRIzol® Reagent was substituted for lysis buffer provided in extraction kit. (2) An on-column DNase digestion step was performed between RW1 washes. RNA was quantified using the Nandrop ND-1000 Spectrophotometer (NanoDrop Technologies Inc, Wilmington, DE, USA). 1 µg RNA was reverse transcribed using SuperScript III reverse transcriptase enzyme (Invitrogen, Carlsbad, CA, USA). cDNA was analysed by real-time quantitative PCR (RQ-PCR) using the ABI Prism 7000 (Applied Biosystems, Warrington, UK). TaqMan ® Gene Expression Assays were used in TaqMan ® Universal Mastermix (Applied Biosystems). Assays targeting 7 genes were as follows: NIS, RARα, RARβ, ERα, PI3K, THRα and THRβ. Expression data was normalised to corresponding values for endogenous control assays for Mitochondrial Ribosomal Protein L19 (MRPL19) and Peptidyl-Prolyl Isomerase A (PPIA) [24]. The comparative ΔΔCt method was used to calculate relative gene expression and expressed as a Log_10_ Relative Quantity [25]. Data was expressed relative to the lowest detectable sample in the cohort analysis and to appropriate diluent controls in the cell line stimulation study.

### Immunohistochemistry

5 µm sections of snap frozen normal, fibroadenoma and malignant breast biopsies from a subset of the same patient cohort were cut and mounted on slides. These sections were probed using a mouse anti-human NIS antibody [26], and the Discovery™ DABMap Kit on a Discovery™ Ventana system. Slides were washed in warm soapy water to remove oil, followed by rinsing in ddH_2_0. The slides were then treated sequentially as follows: 3 mins in 75% alcohol, 3 mins in 95% alcohol, 3 mins in 100% alcohol, and 2 serial immersions of 3 mins in Xylene. The slides were then mounted in DPX mounting medium and analysed using an Olympus BX60 microscope and analySIS Soft Imaging System. Images were captured at 200× and 400× magnifications.

### Cell Culture

T47D cells, characterised as estrogen and progesterone receptor (ER/PR) positive and Her-2/neu (Her2) negative, were cultured in RPMI 1640 medium. SK-Br-3 cells (ER/PR –ve, Her-2/neu +ve) were cultured in McCoys-5a medium. All media were supplemented with 10% foetal bovine serum (FBS), 100 IU/ml penicillin/100 µg/ml streptomycin (P/S) and 1% l-glutamine. Cells were incubated at 37°C and 5% CO_2_, with media change three times per week and passage every seven days.

### In vitro stimulation of NIS gene expression

In initial experiments, performed in triplicate, T47D and SK-Br-3 cell lines were seeded at a density of 2.4×10^4^/cm^2^, and incubated in the presence of β-Estradiol (E_2_)(1 nM, 10 nM, 50 nM), all trans Retinoic Acid (ATRA, 0.1 µM,1 µM,5 µM) or L- Thyroxine (T_4_)(0.1 µM, 0.5 µM, 1 µM) for 24 or 72 hrs. Media and stimulants were replenished at 24 hr intervals, and control cells were cultured in the appropriate diluent used for each stimulant e.g. 0.1% NH_4_OH for T_4_ and 0.5% DMSO for ATRA.

Following establishment of the optimal time point (24 hrs) and concentration (10 nM E_2_, 1 µM ATRA and 0.1 µM T_4_) the experiment was repeated in triplicate using the optimal concentration individually or in combination. Cells were harvested at the appropriate time points by trypsinisation, centrifuged @ 1000 rpm for 4 mins and the cell pellet stored @ −80°C. RNA was extracted and the corresponding cDNA analysed using RQ-PCR targeting NIS and the endogenous controls PPIA and MRPL19 as described. The data was expressed relative to cells grown in appropriate diluent controls.

### Statistical analysis

Continuous variables are summarised numerically by Mean (SEM), and graphically using boxplots and scatterplots. A two sample Student's t test and a general linear model ANOVA were used to compare mean responses as appropriate. Scatter plots with linear regression and Lowess smoother lines were used to determine the likely relationships between variables of a population. The degree of relationship was assessed using Pearson correlation coefficients. The corresponding 1-tailed p value was calculated using the r to p value calculator available at http://faculty.vassar.edu/lowry/tabs.html.

## Results

### NIS expression in human breast tissue

Total RNA extracted from malignant (n = 75), normal (n = 15), and fibroadenoma (n = 10) breast tissue biopsies was analysed using RQ-PCR targeting NIS, RARα, RARβ, ERα, PI3K, THRα and THRβ. The resulting gene expression data was normalised to endogenous controls MRPL19 and PPIA and expressed relative to the lowest detectable sample for each target. Results were expressed as Log_10_ Relative Quantity (RQ).

**Figure 1:**
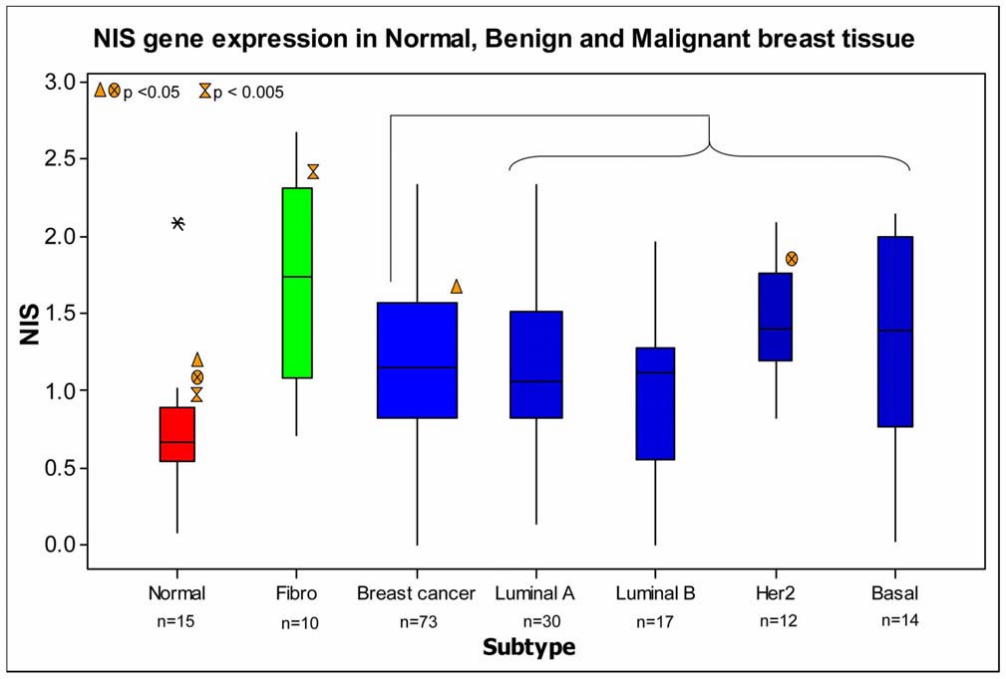
NIS expression in normal, fibroadenoma (“Fibro”) and malignant breast tissue. Results have been normalised to the endogenous controls PPIA and MRPL19 and expressed relative to the lowest detectable sample. Note: Results in breast cancer patients are also stratified by epithelial subtype.

NIS was detectable in 98/100 samples analysed. Expression levels were significantly higher in breast cancer (Mean (SEM) 1.18 (0.07) Log_10_ RQ, p<0.05) and fibroadenoma tissue (1.69 (0.21) Log_10_ RQ, p<0.005) than normal tissue (0.70 (0.12) Log_10_ RQ, Figure 1). The highest level of NIS expression overall was observed in fibroadenoma tissue. When divided on the basis of epithelial subtype, the highest level of NIS expression was observed in the Her2 subgroup (1.43 (0.11) Log_10_ RQ, Figure 1). Data was also subdivided on the basis of prognostic indicators such as disease stage, tumour grade, menopausal status, estrogen, progesterone, and Her-2/neu status, with no statistically significant relationship observed between NIS and these clinical characteristics.

### Immunohistochemical analysis of NIS protein in selected human breast tissues

**Figure 2:**
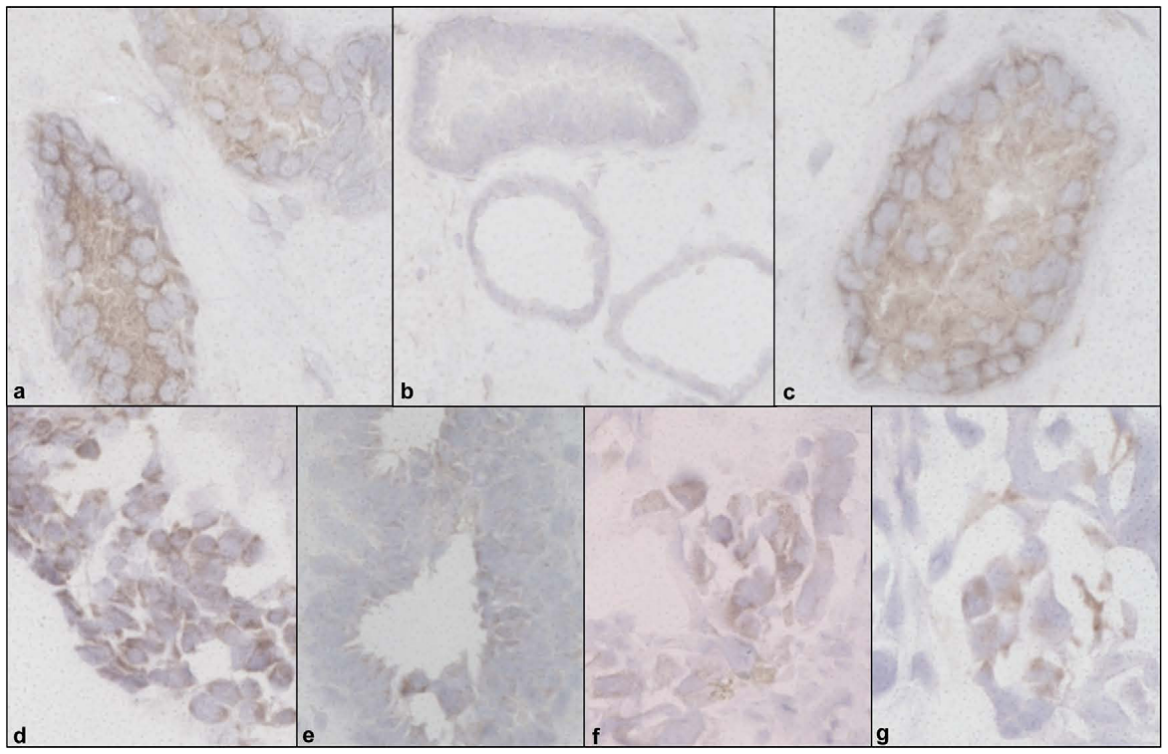
Immunohistochemical detection of NIS protein expression in 5 µM sections of selected tissues from patient cohort. (**a**) Thyroid tissue (positive control) (400×), (**b**) Antibody free Fibroadenoma (negative control) (200×), (**c**) Fibroadenoma (400×). Also shown are breast cancer epithelial subtypes Luminal A (**d**), Luminal B (**e**), Her2 (**f**) and Basal (**g**) at 400× magnification.

Tissue sections of selected snap frozen biopsies from the patient cohort were prepared and examined for expression of NIS protein using a mouse anti human monoclonal NIS antibody and DABMap staining. Thyroid tissue, which was used as a positive control, was strongly immunopositive for the protein (Figure 2a). Antibody free controls for each sample were confirmed to be negative, with an example of the fibroadenoma antibody free control shown (Figure 2b, Note: 200× magnification used for a larger field of view). Strong membrane targeted NIS expression was observed in fibroadenoma tissue (Figure 2c). Positive staining was also observed in breast cancer tissues with an example shown of each individual epithelial subtype; Luminal A (Figure 2d), Luminal B (Figure 2e), Her2 (Figure 2f) and Basal (Figure 2g).

### Expression of receptors for putative NIS regulators in human breast tissue

**Figure 3:**
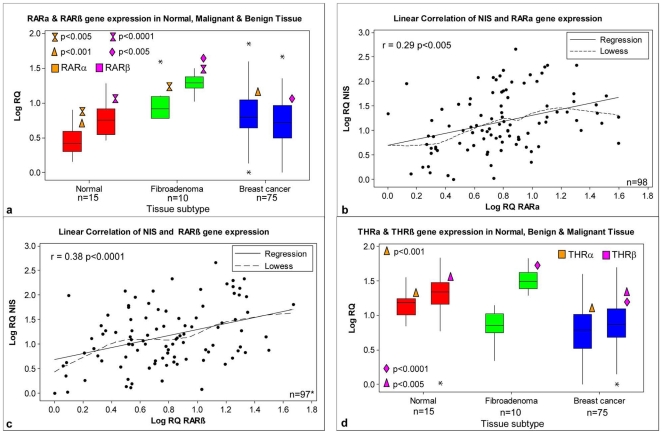
RARα and RARβ expression in breast tissue samples, and their relationship with NIS. (**a**) RARα and RARβ expression in normal, malignant and fibroadenoma breast tissue. Results have been normalised to the endogenous controls PPIA and MRPL19 and expressed relative to the lowest detectable sample. Correlations of NIS gene expression with RARα (**b**) and RARβ (**c**) with r and p values shown (* one outlier removed). (**d**) THRα and THRβ expression in normal, malignant and fibroadenoma breast tissue.

Expression of both RARα and RARβ were highest in fibroadenoma tissue (Figure 3a). In the case of RARβ, this was significantly higher than both normal and malignant tissue (p<0.001, p<0.005 respectively Figure 3a), which displayed similar levels of RARβ expression. In the case of RARα, expression was also significantly higher in tumour compared to normal tissue (p<0.005, Figure 3a), with no significant difference between malignant and fibroadenoma tissue levels (Figure 3a). Using scatter plots with linear regression and Lowess smoother lines, linear relationships were observed between NIS and both retinoic acid receptors (Figure 3b, 3c). A Pearson Correlation Coefficient of r = 0.29 was observed between NIS and RARα (p<0.005, Figure 3b), while a more robust relationship was observed between NIS and RARβ (r = 0.38, p<0.0001, Figure 3c).

In the case of thyroid hormone receptors, expression of both isoforms (α and β) was significantly lower in malignant compared to normal tissue (p<0.001, p<0.005 respectively, Figure 3d). Conversely, THRβ was significantly higher in fibroadenoma tissue compared to normal (p<0.0001). No significant relationship was observed between NIS and THR α or β in the complete cohort.

**Figure 4:**
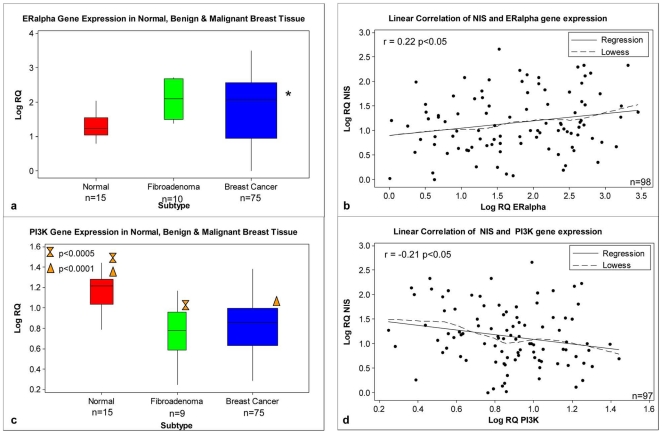
ERα and PI3K expression in breast tissue samples and their relationship with NIS. Expression of ERα (**a**) and PI3K (**b**) in normal, malignant and fibroadenoma breast tissue. Results have been normalised to the endogenous controls PPIA and MRPL19 and expressed relative to the lowest detectable sample. Correlation of NIS gene expression with ERα (**c**) and PI3K (**d**) with r and p values shown.

ERα expression was higher in both fibroadenoma and malignant breast tissue compared to normal (Figure 4a). A linear relationship and significant correlation was observed between NIS and ERα (r = 0.22 p<0.05, Figure 4b). This was particularly strong within the Her2 epithelial subtype (r = 0.76 p<0.01, n = 13). PI3K expression was significantly lower in both fibroadenoma and malignant compared to normal breast tissue (p<0.0005, p<0.0001 respectively, Figure 4c) with a negative correlation observed between NIS and PI3K (r = −0.21 p<0.05, n = 97, Figure 4d).

### In vitro stimulation of NIS gene expression

Following on from the gene expression study in the breast tissue samples, some of the observations were further investigated in an in vitro setting through stimulation of breast cancer cell lines with ligands targeting putative regulators of NIS. Breast cancer cell lines, grown in the presence of varying concentrations of estradiol (E_2_), all trans retinoic acid (ATRA) and thyroxine (T_4_) for 24 or 72 hrs, were harvested and changes in NIS gene expression analysed by RQ-PCR. Expression data is normalised to endogenous controls PPIA and MRPL19 and expressed relative to cells grown in appropriate diluent controls.

**Figure 5:**
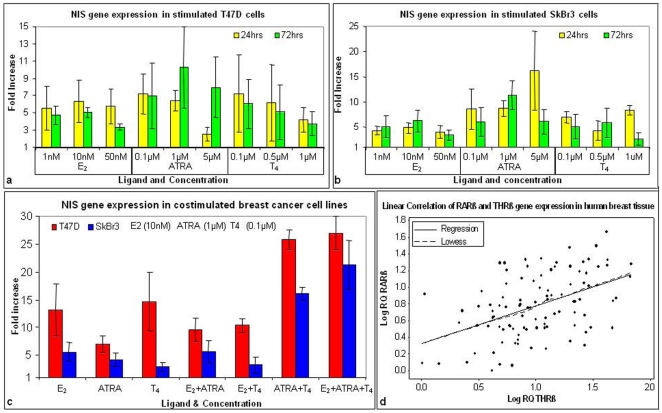
Effect of β-estradiol (E_2_), all trans Retinoic Acid (ATRA) and L-thyroxine (T_4_) on NIS expression in breast cancer cell lines. Effect of indivdual stimulants on NIS expression in (**a**) T47D and (**b**) SkBr3 cell lines. Results have been normalised to the endogenous controls PPIA and MRPL19 and expressed relative to diluent controls, with the baseline representing 2^−(ddCt diluent control)^. (**c**) NIS expression in T47D and SkBr3 cells following 24 hr stimulation with optimal concentrations of ligands alone and in combination. (**d**) Correlation of RARβ with THRβ gene expression within patient cohort with r and p values shown.

In the case of the T47D cell line NIS expression was increased in the presence of all 3 ligands following both 24 and 72 hr incubation (Figure 5a): E_2_ (range 3–6 fold), ATRA (range 3–10 fold), T_4_ (range 4–7 fold). The greatest, most consistent increases seen in T47D cells were with 10 nM E_2_ at 24 hrs (Mean (SEM) fold change 6 (2) fold), 1 µM ATRA at 24 hrs (6 (1) fold), and 0.1 µM T_4_ at 72 hrs (6 (3) fold).

A similar pattern was observed for the SK-Br-3 cell line (Figure 5b). Stimulation with E_2_ resulted in a 3–7 fold increase in NIS expression, ATRA stimulated a 6–16 fold increase, while T_4_ resulted in a 3–8 fold increase. The greatest most consistent change in NIS expression was observed in the presence of 10 nM E_2_ at 24 hrs (Mean (SEM) fold change 5 (1) fold), 1 µM ATRA at 24 hrs (9 (2) fold), and 0.1 µM T_4_ at 24 hrs (7 (1) fold).

As shown in Figure 5a and 5b, incubation in the presence of any of the stimulants for 72 hrs did not confer a significant advantage and so subsequent incubations were performed for 24 hrs.

Optimal concentrations of ligands were selected based on the largest most consistent increase in NIS expression observed in initial experiments, i.e. 10 nM E_2_, 1 µM ATRA and 0.1 µM T_4_. The experiment was repeated in triplicate treating cells with ligands for 24 hrs both individually and in combination.

Increased NIS expression was observed in the presence of all 3 ligands when used individually (Figure 5c). In both the T47D and Sk-Br-3 cell lines, the combination of ATRA and T_4_ created the greatest increase in NIS expression observed (Mean (SEM): 26 (1) and 16 (1) fold increase respectively, Figure 5c). Addition of E_2_ to this combination did not have a significant impact on NIS gene expression. The change in expression was greater than the sum of increases when the cells were cultured in the presence of each factor individually. Based on this, gene expression data from the patient cohort was re-examined to determine whether there was any relationship between retinoic acid receptors and thyroid hormone receptors in human tissue samples. Further investigation of the expression of these receptors in human tissues revealed a linear relationship and significant positive correlation between RARβ and THRβ across all samples (r = 0.49, p<0.0001, n = 100, Figure 5d).

## Discussion

The presence, function and regulation of the sodium iodide symporter have been well established in thyroid tissue. This has led to a better understanding of thyroid diseases and effective imaging and therapy using radioactive iodide [27]. NIS is also known to play a role in the breast and is present in a functional capacity in lactating breast tissue [2]. Expression of the NIS gene and functional protein has been demonstrated in fibroadenoma and malignant breast tissue but typically not in normal breast tissue [3], [6], [11]. Understanding the nature of mammary NIS expression would not only be useful in breast cancer research but may potentially benefit understanding of other extrathyroidal malignant tissues expressing NIS, including ovary, lung, colon, and endometrium [6]. Investigation of mammary NIS regulation will support elucidation of the nature of NIS dysregulation in malignant compared to normal tissue.

To date, quantitative analysis of NIS expression in a large cohort of human breast tissue samples has not been performed. In the current study, expression of NIS and receptors for putative regulators was quantified by RQ-PCR, in 100 human breast tissue samples, and relationships observed further investigated in vitro. NIS expression was observed to be significantly higher in malignant compared to normal breast tissue, with the highest levels detected in fibroadenoma. This data agrees with previous smaller studies showing increased NIS expression in malignant tissue [3], [4], and also more surprisingly in fibroadenoma compared to malignant tissue [3]. Immunohistochemical analysis of a subset of tissues from the same cohort also confirmed the presence of NIS protein in selected malignant and benign breast tissues. Thus it is abundantly clear that native NIS expression in breast cancer cannot serve as a useful marker of malignancy.

NIS expression levels across all tissue samples examined were shown to correlate to that of receptors for potential regulators highlighted previously in breast cancer cell lines and animal models [2], [13], [17], [18].

Expression of NIS, RARα and ERα were significantly higher in malignant compared to normal breast tissue, with a significant positive correlation observed between NIS and RARα (r = 0.29 p<0.005) and ERα (r = 0.22 p<0.05). This supports previous in vitro and in vivo studies showing ATRA mediated stimulation of NIS expression [13], [17]. Alotaibi and colleagues recently reported a potent enhancer of NIS expression located in the first intron of the NIS gene that mediates direct regulation by trans retinoic acid-stimulated nuclear receptors which include RARα [28]. The same group also reported an estrogen-responsive element in the promoter region of NIS gene that specifically binds ERα and mediates ERα-dependent activation of transcription [18].

Previous studies using breast cancer cell lines have suggested that selective stimulation of RARβ increases NIS expression more efficiently than that of RARα [17]. The data presented here on expression of RARβ and NIS in human breast tissue samples supports this, with the largest, most significant correlation detected between expression of these two genes (r = 0.38 p<0.0001).

PI3K was previously reported to mediate increased NIS gene expression in MCF-7 breast cancer cells [19], [29]. However, in the current study an inverse relationship and significant negative correlation between NIS and PI3K was observed (r = −0.21 p<0.05). This negative correlation suggests that inhibition of PI3K may increase mammary NIS expression. Stratification of the patient cohort based on tumour epithelial subtype revealed that while the overall negative correlation between NIS and PI3K was significant, within the Luminal A subtype which MCF-7 cells represent, there was a positive relationship (albeit not significant), between the two genes. This may explain the conflicting data observed and highlights the importance of using multiple cell types when attempting to represent this heterogeneous disease.

It is interesting to note that the 3 genes in this study found to be differentially expressed in fibroadenoma and malignant tissue (NIS, RARβ and THRβ), have all been suggested to have tumour suppressor roles. Gol Choe et al, have proposed NIS transgene inhibition of oncogenic RAS-mediated signalling [30]. RARβ2 overexpression has been shown to result in decreased metastatic potential of MDA-MB-435 in vivo [31]. Reports of decreased THRβ expression in breast tumours relative to normal tissue [21], [23], [32], which was also observed in the current study, and THRβ1 suppression of tumour invasiveness and metastasis [21] suggest an anti-tumour role for THRβ. Based on these studies, and the data presented here, it may be postulated that in rapidly proliferating mammary epithelial cells, loss of expression of these genes may be involved in tumour pathogenesis.

The current study revealed upregulation of NIS expression through stimulation of both the retinoic acid and estrogen receptors with relevant ligands (ATRA and E_2_) in breast cancer epithelial cell lines. Data generated from the ER positive T47D cell line supports previous studies which demonstrated retinoic acid [13], [17] and estradiol stimulation of NIS expression [2], [18]. However, it is interesting that stimulation of NIS gene expression could also be seen using ATRA and E_2_ in the Sk-Br-3 cell line which are characterised as ER negative by IHC. This contradicts the hypothesis that RA-responsive NIS expression is confined to estrogen receptor-alpha (ERα) positive cells [18], and suggests that in the absence of substantial ER expression some other mechanism of stimulation is at play.

While no direct relationship between expression of NIS and THRα or THRβ was observed, in vitro studies revealed significant stimulation of NIS expression in cell lines exposed to thyroid hormone. Dimerization between retinoic acid receptors and thyroid hormone receptors is known to occur [33], [34]. Heterodimerisation between retinoid-X-receptor and pregnane-X-receptor has been identified as the basis for carbazmazepine stimulation of NIS expression in MCF-7 cells [12]. Indeed in the current study, the greatest induction of NIS expression was seen in the presence of ATRA and T_4_ combined, where a synergistic increase greater than the sum of that seen with the individual factors alone was observed. Further analysis of this relationship in patient samples revealed a significant positive correlation between RARβ and THRβ (r = 0.49 p<0.0001). The positive effect on mammary NIS expression observed in vitro is in contrast to the inhibitive role T_4_ plays in thyroidal NIS regulation.

### Conclusion

Although NIS expression is significantly higher in malignant compared to normal breast tissue, the highest level was detected in fibroadenoma, indicating that NIS expression in breast cancer cannot serve as a useful marker of malignancy. However this does not preclude the use of native NIS expression as a tool for imaging or therapy of the disease. Indeed, a pilot imaging study is currently underway to determine radioiodine accumulation and dosimetry in breast cancers using ^124^I PET/CT (Clinicaltrials.gov). In the context of other non-thyroidal cancers, a previous Phase I trial demonstrated safety and feasibility of virus mediated delivery of NIS for imaging prostate cancer [35]. Two further clinical trials are also currently underway utilising NIS as both a reporter for virus tracking in patients with multiple myeloma, and for gene therapy of prostate cancer in conjunction with radioiodine (Clinicaltrials.gov).

The data presented support a role for retinoic acid and estradiol in mammary NIS regulation in vivo, and also highlight potential thyroidal regulation of mammary NIS mediated by thyroid hormones. Increased understanding of extra-thyroidal expression and regulation of NIS will support further development of the imaging and therapeutic potential of NIS in the clinical setting.
